# Perceptions of the ankle brachial index amongst podiatrists registered in Western Australia

**DOI:** 10.1186/1757-1146-5-19

**Published:** 2012-07-30

**Authors:** Pamela Y Chen, Kaye M Lawford, Naiya Shah, Julie Pham, Virginia M Bower

**Affiliations:** 1 Podiatric Medicine Unit, School of Surgery, Faculty of Medicine, Dentistry and Health Sciences, The University of Western Australia, 35 Stirling Hwy, Crawley, WA, 6009, Australia

## Abstract

**Background:**

The ankle brachial index (ABI) is an objective diagnostic tool that is widely used for the diagnosis of peripheral arterial disease. Despite its usefulness, it is evident within the literature that many practitioners forgo using this screening tool due to limiting factors such as time. There is also no recommended technique for ABI measurement. The purpose of this study is to investigate the perceptions of the use of ABI clinically among Western Australian podiatrists.

**Methods:**

This study was a cross sectional survey which evaluated the perceptions of the ABI amongst registered podiatrists in Western Australia. The study sample was obtained from the register of podiatrists listed with the Podiatrists Registration Board of Western Australia. Podiatrists were contacted by telephone and invited to participate in a telephone questionnaire. Chi-square tests were performed to determine if there was a statistically significant relationship between use of the ABI and podiatrists’ profile which included: sector of employment; geographical location; and length of time in practice.

**Results:**

There is a statistically significant relationship (p=0.004) between podiatrists’ profile and the use of ABI, with higher usage in the tertiary hospital setting than in private practice. Length of time spent in practice had no significant impact on ABI usage (p=0.098). Time constraints and lack of equipment were key limiting factors to performing the ABI, and no preferred technique was indicated.

**Conclusion:**

Western Australian podiatrists agree that the ABI is a useful tool for lower limb vascular assessment, however, various factors influence uptake in the clinical setting. This study suggests that a podiatrists’ profile has a significant influence on the use of the ABI, which may be attributed to different patient types across the various settings. The influence of time spent in practice on ABI usage may be attributed to differences in clinical training and awareness of lower limb pathology over time. The authors recommend publication of ‘best practice’ guidelines to ABI performance, as well as further education and financial rebates from health organizations to facilitate increased utility of the ABI based on the findings of this study.

## Background

Peripheral arterial disease (PAD) is a systemic manifestation of atherosclerosis characterized by atherosclerotic occlusive disease of the lower extremity. PAD is associated with several podiatric implications such as gangrene, lower extremity ulcers and amputations, especially in the diabetic population [1,2]. Although the most recognizable symptom of PAD in the lower limb is intermittent claudication, up to 90% of people with PAD are asymptomatic or experience atypical symptoms especially in the early stages of the disease [1]. In light of these debilitating complications, there is a need for a simple assessment that can function as an effective tool to detect PAD, such as the ABI [3].

The ABI provides an indication of the functional status of circulation in the lower extremity by calculating the ratio of the ankle and brachial systolic blood pressures [1,4]. It is obtained by a simple and non-invasive procedure involving measurement of a patient’s systolic blood pressures at both dorsalis pedis (DP) and tibialis posterior (TP) arteries in both feet as well as the brachial artery in both arms [1]. The ratio is considered to be of high diagnostic value, with an ABI of <0.9 associated with an increased risk of cardiovascular events such as coronary heart disease, stroke and death [1,2,5].

In addition, the ABI has been compared to angiography, which is the gold standard for arterial assessment in the lower limb. Previous studies have identified a sensitivity of 95% and specificity of at least 99% when compared against angiography [2,6]. This has led to the American Heart Association acknowledging the ABI as having 90% sensitivity and 98% specificity for the detection of stenosis of arteries in the lower limb [1,2,7,8]. The ABI is also considered a key diagnostic test in the Trans-Atlantic inter-society consensus for the management of PAD [6].

Whilst the literature is in agreement with regards to the usefulness of the ABI, there is a lack of consensus regarding the most reliable method used for its calculation. According to the literature, there are three methods of obtaining a brachial pressure value (for use in the denominator) and five ways of obtaining a pedal pressure value (for use in the numerator). Whilst Caruana et al. [9] identify that the higher of the two pressures is conventionally used, McDermott et al. [10] suggested that the lower of both pedal values is most predictive of objective measurement of lower limb vascular function.

Furthermore, despite the demonstrated usefulness of the ABI in the literature, two studies conducted in the United States of 261 and 620 primary health care practitioners respectively, revealed limited use of the ABI in the primary clinical setting [11]. The studies identified that 69% and 67% respectively never used the ABI as a screening tool for the detection of PAD [11]. Mohler et al. [11] attributed this to several factors, including the lack of training, staff availability and equipment, patient willingness, clinical significance as well as financial and time constraints, with time constraint found to be the most significant limitation. For these findings to be validated, more extensive studies are required, particularly in the field of podiatry due to the important role the podiatrist plays in the identification and management of lower limb pathology.

Based on the observed discrepancies of the use of ABI in the literature, the purpose of this study was to identify the perceptions Western Australian podiatrists hold towards the ABI. Study objectives included identifying differences in techniques, limitations of its use, and possible alternatives to assess lower limb vascular status. It is anticipated that the identification of these factors and influences will serve as a platform for future research or continuing education programs to ensure that the podiatry community has an adequate evidence-based understanding of the use of the ABI.

## Methods

The aim of this study was to evaluate the perceptions of the ankle brachial index amongst podiatrists in Western Australia. Objectives of this study were to identify:

1. underlying factors contributing to the use, or lack of use of the ABI in the clinical setting including sector of employment; podiatrists geographical location; and length of time in practice,

2. variation in technique used to perform the ABI,

3. methods used to calculate the ABI,

4. barriers/limitations to using the ABI,

5. alternatives used to evaluate vascular status other than the ABI.

The study population included all registered podiatrists listed on the Podiatrists’ Registration Board of Western Australia Website. The study sample was registered podiatrists who were successfully contacted during the data collection period and agreed to participate. The study design was a cross-sectional survey delivered via a scripted telephone questionnaire. Restrictions of time, cost and public availability of podiatrists’ telephone contact details on the World Wide Web, were limitations which had to be managed within the scope of the project [12].

The names of all 317 podiatrists registered in Western Australia were downloaded from the now obsolete Podiatrists’ Registration Board of Western Australia in March 2010 (The State Registration Boards have since been replaced by the nation-wide Australian Health Practitioner Regulation Agency). Contact details of these 317 podiatrists were sourced from various sources as listed below:

1. www.findapodiatrist.org (a website where all member podiatrists of the Australian Podiatry Associations are available)

2. www.yellowpages.com.au (search function “Podiatrists” in “Western Australia”)

3. www.whitepages.com.au (search function “Podiatrists” in “Western Australia”)

Due to restrictions in time the researchers were only able to spend two months sourcing telephone details and making contact with all registered podiatrists.

The authors identified that many podiatrists work in different sectors of health and often in more than one geographical area. As a consequence, all participants were given the opportunity to represent information related to either, or both of their work sectors. Participants choosing to respond only once were invited to do so based on the sector of work they spent most of their time in. Data pertaining to this variable was not recorded and therefore analysis of this data was not performed.

Ethics approval was obtained from the University of Western Australia Human Research Ethics Committee. All podiatrists whose contact details were obtained from the various sources mentioned previously were contacted, and a scripted telephone introduction to the study was read, inviting the podiatrist to participate or decline participation in the study. If verbal consent was obtained, the questionnaire was then read with all responses recorded on an excel spreadsheet.

All participants were advised that all information would be de-identified and kept strictly confidential.

### Questionnaire outline

The questionnaire tool developed for this study was long and hence only a summary of the tool will be provided below. The questionnaire was divided into four sections. The first section identified the podiatrists’ profile which included: sector of employment, geographical location of practice and the number of years he/she has been practicing.

The questions in the second section were based on identifying specific perceptions of the ABI. Participants were first asked if they considered the ABI a useful tool for the vascular assessment of the lower limb, following which they were presented with various scenarios and asked if these influenced their decision to perform an ABI. These scenarios were:

1. palpability of pulses,

2. doppler waveform results,

3. intermittent claudication.

Podiatrists who indicated that they used the ABI were then requested to describe the technique routinely used in section three of the questionnaire. Open-ended responses were matched to a list of choices available to the researchers which are listed below:

1. the position of the patient,

2. the length of time a patient spent lying down prior to ABI measurement, if applicable,

3. the number of arteries from which the brachial pressure(s) were measured,

4. the number of arteries from which the pedal pressure(s) were measured,

5. the values used to calculate the ABI,

6. the instruments used to perform the ABI.

These categories were selected based on variables identified by Lange et al. [8] and Caruana etal. [9], and further refined in consultation with a number of experts in the area of the high risk foot.

The final section was open ended and designed to identify common limiting factors to performing the ABI as well as alternatives used in lower limb vascular assessment. All responses were written down verbatim. From these, the authors identified common themes and grouped the responses accordingly.

### Data analysis

A combination of descriptive and inferential analysis was used in this study. IBM SPSS Statistics version 19 was used to perform statistical analysis of the study data. For objective one, Chi-square tests were performed to identify if a significant relationship existed between the dependent variable ABI and the independent variables: sector of employment; geographical location and; length of time in practice. A *p*- value threshold of 0.050 was used to determine the level of significance between variables. Descriptive statistics were performed to identify common themes for study objectives two to five.

## Results

### Response rate

Of 317 podiatrists registered with the Podiatrists’ Registration Board of Western Australia, contact details of only 110 podiatrists were successfully obtained during the two-month window allocated for data collection. Of these, 18 declined participation in the study giving a response rate of 83.6%. In addition, 13 podiatrists chose to provide multiple responses to the questionnaire based on the different sectors of health they worked in.

### Podiatrists’ profile

Seventeen out of 18 (94.0%) podiatrists practicing in tertiary hospitals indicated that they used the ABI, as compared to 35 out of 68 (51.5%) private practitioners, with varied usage rates amongst podiatrists practicing in community health and secondary hospital settings. This suggests that podiatrist sector of employment influences his or her use of the ABI, with a *p*-value of 0.004 as seen in Table 1.

**Table 1:** Podiatrists’ sector of employment and ABI utility

**Sector of employment**	**Uses ABI**	**Does not use ABI**	**( *p* -value)**
**Private practice**	35 (51.5%)	33 (48.5%)	0.004
**Community health**	8 (66.7%)	4 (33.3%)	
**Secondary hospital**	6 (85.7%)	1 (14.3%)	
**Tertiary hospital**	17 (94.4%)	1 (5.6%)	

Responses to this study also indicated that geographical area had no impact on a practitioners’ use of the ABI, with 50 out of 78 (64.1%) podiatrists practicing in the metropolitan area and 16 out of 27 (59.2%) podiatrists practicing in rural Western Australia indicating use of the ABI. After statistical analysis it was found that there was no significant difference between the two responses with a *p*-value 0.419 as seen in Table 2.

**Table 2:** Podiatrists’ geographical location and ABI utility

**Location of employment**	**Uses ABI**	**Does not use ABI**	**( *p* -value)**
**Metropolitan**	50 (64.1%)	28 (35.9%)	0.419
**Rural**	16 (59.2%)	11 (40.8%)	

Finally, the length of time a podiatrist has spent in practice was found to have no significant impact on his or her use of the ABI. New graduates were just as likely to perform the ABI as their more experienced colleagues. The *p*-value for the relationship between these two variables was 0.098 as seen in Table 3.

**Table 3:** Length of time in practice and ABI utility

**Length of time in practice**	**Uses ABI**	**Does not use ABI**	**( *p* -value)**
**< 5 years**	12 (85.7%)	2 (14.3%)	0.098
**Between 5 and 10 years**	16 (69.6%)	7 (30.4%)	
**Between 11 and 15 years**	15 (65.2%)	8 (34.8%)	
**>16 years**	23 (51.1%)	22 (48.9%)	

### Technique of ABI measurement

#### Number of arteries from which the brachial and pedal pressure(s) were measured

An equal number of podiatrists indicated measuring pressure from one or both brachial arteries. However, there was a greater variation in the responses received for the number of arteries from which pedal pressures were measured. Twenty-four (36.3%) podiatrists indicated only measuring pressure from one foot, eight (12.1%) responses were for both arteries on one foot, and nine (13.6%) and one (1.5%) responses were given for pressures from one PT and one DP artery respectively. Forty-eight (72.7%) podiatrists indicated assessing pressures on both feet, with 22 (33.3%) using all four pedal arteries, 19 (28.8%) using both PT arteries and seven (10.6%) using both DP arteries.

#### The values used to calculate the ABI

The selection of brachial and pedal pressures for calculation of the ABI was also varied. Of the 33 podiatrists who indicated measuring pressures from both brachial arteries, seven (21.2%) used the average of both pressures, 12 (36.4%) used the highest of both pressures, one(3.0%) used the lowest of both pressures and 13 (39.4%) used both separately, obtaining a separate index for both left and right feet.

Thirty-three podiatrists indicated only using the pressure obtained from the PT in calculation of the ABI. Of these, 28 (84.8%) measured only pressures from PT arteries (one or both limbs), whereas five (15.2%) used the PT value regardless of other pressure values obtained. Similarly, nine podiatrists indicated using the pressure obtained from the DP, of which eight (88.9%) only measured pressures from DP arteries and one (11.1%) who used the DP value in calculation regardless of other results.

Of the eight podiatrists who obtained pressures from both arteries on one foot, four (50.0%) indicated using the highest and three (37.5%) indicated using the average of both values in calculation of the ABI. One (12.5%) podiatrist only used the value from the PT artery despite obtaining pressures from the DP as well.

Twenty-two podiatrists indicated using pressures from all four pedal arteries, with 14 (63.6%) using the highest, four (18.1%) each using the lowest and average values and four (18.1%) using all four arteries, obtaining two ABI values per foot.

### Limitations and alternatives to performing the ABI

**Figure 1:**
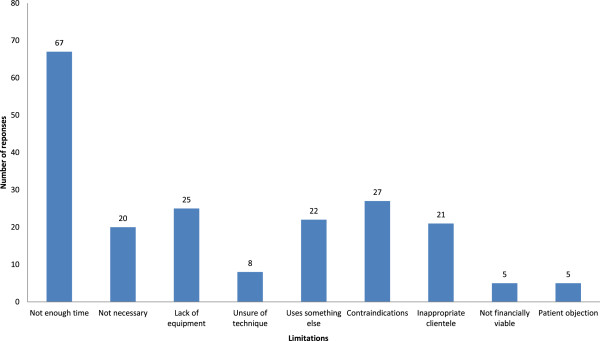
Limitations to performing the ABI.

Sixty-seven podiatrists indicated that the lack of time was a key limiting factor in their use of the ABI. Patient contraindications (27 responses) were the next most common, followed by lack of equipment (25 responses) and the use of alternative tests (22 responses). Of interest, five responses indicated a lack of financial viability in performing the ABI, and another five indicated that their patients routinely objected to having the ABI performed. The distribution of the responses received can be seen in Figure 1.

**Figure 2:**
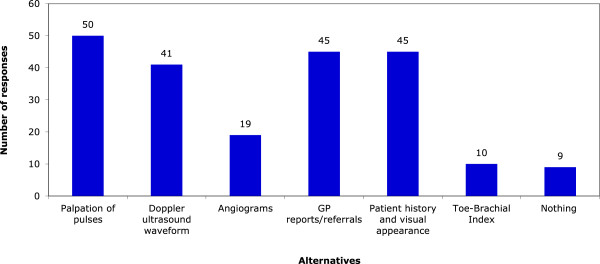
Alternatives to performing the ABI.

Several alternatives to lower limb vascular assessment were also proposed. The most frequent alternative used was palpation of pulses (50 responses), followed by the visual appearance of the limb and the patients’ medical history (45 responses) and correspondence from other health practitioner (usually GPs) detailing the patients’ vascular history (45 responses). The distribution of all the proposed alternatives can be seen in Figure 2.

## Discussion

### Incidence and use of ABI

The ABI is an objective diagnostic tool that is widely recognized and recommended as a useful test for the identification of PAD, with a reported sensitivity and specificity of 95.0% and 99.0% respectively. Despite this, it is striking to notice that a large number of clinicians do not regularly use the ABI. This is demonstrated by a study conducted by Mohler et al. on physicians and non-physician primary care professionals. Mohler et al. [11] found a reported incidence of use of 31.0% in a study population of 263 practitioners and 33.0% in a population of 623 practitioners. In the current study, the first of its kind conducted within the scope of podiatry in Western Australia, 62.9% of podiatry practitioners indicated regular usage of the ABI. This higher rate to uptake may be explained by the fact that podiatrists are more aware of the value of the ABI due to the nature of podiatry practice which is focused on lower extremity pathology. It would be interesting to compare the rate of uptake of the ABI among other podiatry cohorts outside of the Western Australian context to see how the Western Australian data compares [11].

In addition, this study identified that the clinical setting in which a podiatrist works influences his or her use of the ABI. Results of this study indicated that 94.0% of podiatrists practicing in tertiary hospitals utilise the ABI whereas only 51.1% of podiatrists practicing privately use the ABI as part of their clinical assessment. The authors hypothesize that this is due to the different clientele with patients of a higher acuity seen in the tertiary hospital setting as opposed to in private practice.

It was thought that a difference in accessibility to specialist vascular services between rural and metropolitan locations would contribute to a higher usage of the ABI in the rural Western Australia, however, this was not apparent in the results of this study. This may be due to the influence of other factors such as acuity of patients and other limiting factors unique to rural settings, which were not investigated in this study.

The findings of this study also indicate no significant relationship between the length of time spent in practice and a podiatrists’ use of the ABI. This relationship has not been previously analyzed in the literature; however, our study findings suggest a consistency in clinical training and awareness of the complications of diabetes and PAD amongst podiatrists irrespective of their years of clinical experience.

### Techniques used in measurement and calculation of the ABI

The consensus conference report from the ‘Prevention Conference V’ defined the ABI as the quotient of the higher of systolic pressures from the two pedal arteries and the average of both pressures from brachial arteries [7]. However, other modes of calculation have been explored and described extensively in the literature [7,8,13]. Assuming pressures from both brachial arteries are measured, three different values can be used in the calculation of the ABI which include the highest, lowest, and average pressures from both arteries. In the foot, pedal pressure values can be obtained in five ways which includes measuring pressures from either the DP or PT arteries, or by using the highest, lowest and average values obtained if pressures are obtained from both arteries [13,14].

There are varying proponents for the different methods ABI measurement and calculation. The usage of appropriate values is significant as it may have implications for the association between the ABI and the underlying burden of atherosclerosis [8,14]. Allison et al. [14] noted that using the lowest pedal pressure provided the best sensitivity and negative predictive value, whilst using the highest pedal pressure provided the best specificity, positive predictive value, and thus overall accuracy for measures of lower limb atherosclerosis. This opinion is echoed by Reed et al. [15] and Schroder et al. [14], with the latter identifying a higher sensitivity associated with a calculation using the lower of two arterial pressures on each limb. Despite the higher test sensitivity of using the lower arterial pressure, using the highest arterial pressure seems more appropriate for evaluating perfusion abnormalities as an abnormal result is usually indicative of more severe disease [14]. However, McDermott et al. [10] was of a contradictory opinion to Allison et al. [14], where they proposed that the lower of both pedal values was most predictive of objective measurement of lower limb vascular function. Lange et al. [8] also supported this view, recommending that the usage of the highest ankle pressure results in the most conservative estimate of PAD prevalence.

While there appears to be a lack of consensus on the best technique, the literature most strongly supports using the lower brachial pressure except when the discrepancy between brachial pressures in both arms exceeds 10 mmHg [7].

The current study reflects the diversity of opinion reported in the literature, which provides no consensus on the most appropriate method to measure and calculate the ABI. However, this study revealed that 50.0% of practitioners were not measuring blood pressures at both brachial arteries and 68.0% were not measuring blood pressures at all four pedal arteries. According to the literature, it is essential for both brachial pressures and all four pedal pressures to be considered, as brachial pressures may be falsely elevated due to subclavian artery stenosis (occurring in up to 20.0% of patients with arterial disease and 4.0% of the normal population) as well as calcification of pedal arteries resulting in falsely elevated pressures [9,13].

The discrepancy seen in the literature is reflected by the findings in this study, and the authors feel that it would be beneficial if a standardized national guideline was developed. The development of clear, evidence based guidelines may be one mechanism to facilitate a greater uptake of use of ABI among practitioners.

### Limitations to and alternatives to performing the ABI

In the study conducted by Mohler et al. [11], limiting factors for practitioners were identified. These include the lack of training, staff availability and equipment, patient willingness, clinical significance as well as financial and time constraints. Of these, time constraint was found to be the most significant limitation [11]. The current study revealed similar findings, with lack of time accounting for a third of all responses out of the nine categories. Lack of equipment was also a significant limiting factor.

Several responses obtained from study participants were surprising. Patient objection is an issue practitioners need to be aware of as even though it is the patients’ prerogative, it is essential that practitioners are aware that patients understand the significance of the ABI and potential complications of undetected PAD. In such cases, it is important that the objection is recognized as an opportunity to provide patient education.

Financial viability was also highlighted as a limiting factor. Potential sources of financial rebate can and should be investigated further. These include the Australian private health fund providers and the Enhanced Primary Care/Chronic Disease Management plan provided through Medicare Australia.

Proposed alternatives used in lower limb vascular assessments were also varied. These included palpation of pulses, doppler ultrasound waveform studies, general practitioner reports and referrals as well as the history and visual appearance of the lower extremity. However, although these are viable tools to assess the vascular status of the lower limb, they do not demonstrate the same sensitivity and specificity as the ABI in the detection of lower limb PAD. This further reinforces the need to develop standardized clinical guidelines to assist practitioners in the timely and accurate assessment of PAD.

### Limitations of the study

The use of a non-validated questionnaire limits the reproducibility and external validity of the study findings. However, to the authors’ knowledge there are no available questionnaires or surveys designed to assess similar parameters amongst podiatric or medical professionals. Should further studies on this topic be conducted on a wider population, development, piloting and validation of a questionnaire is recommended.

The authors also recognize that the brief timeframe allocated to sourcing podiatrists’ contact details restricted the sample population to those whose details were publicly available. For purposes of future research, alternative methods of making contact with all registered podiatrists are recommended. This would give a better representation of the ABI practice and utility amongst the Western Australian Podiatric profession.

It may be difficult to generalize the findings of this study to the broader population of Podiatrists practicing in Australia as the study sample only represented Western Australian Podiatrists. It is possible that there are features of Western Australian podiatrists that are unique from eastern states podiatrists. Considerations such as location of undergraduate training may have an influence on clinical practice and these were not investigated in this study. It should also be noted that only one third of the total 317 podiatrists registered in Western Australia participated in this study.

## Conclusion

This study demonstrated that there are a wide variety of perceptions of the ABI held by podiatrists registered in Western Australia. Despite the usefulness of the ABI as confirmed in the literature, the use of the ABI in clinical practice is relatively limited and this is often attributed to a lack of time and equipment. Different techniques also exist in ABI measurement and calculation. Standardized guidelines and continuing education on the use of ABI is required for the benefit of the podiatry practitioners and their patients.

## Abbreviations

ABI, Ankle brachial index; PAD, Peripheral arterial disease; GP, General practitioners; PT, Posterior tibial artery; DP, Dorsalis pedis artery.

## Competing interests

The authors declare no known competing interests.

## Authors’ contributions

The authors CP, LK, SN and PJ contributed equally to this project. This was done under the guidance and supervision of BV. All authors read and approved the final manuscript.
